# The potential role of abnormal angiotensin-converting enzyme 2 expression correlated with immune infiltration after SARS-CoV-2 infection in the prognosis of breast cancer

**DOI:** 10.18632/aging.203418

**Published:** 2021-08-19

**Authors:** Yufeng Jiang, Ling Chen, Jinsheng Shen, Xiaofei Mei, Jialu Yao, Tan Chen, Yafeng Zhou

**Affiliations:** 1 Department of Cardiology, Dushu Lake Hospital Affiliated to Soochow University, Suzhou, Jiangsu Province, P.R. China; 2 Department of Endocrinology, The First Affiliated Hospital of Soochow University, Suzhou, Jiangsu Province, P.R. China

**Keywords:** COVID-19, SARS-CoV-2, ACE2, immune infiltration

## Abstract

The potential role of abnormal ACE2 expression after SARS-CoV-2 infection in the prognosis of breast cancer is still ambiguous. In this study, we analyzed ACE2 changes in breast cancer and studied the correlation between ACE2 and the prognosis and further analyzed the relationship between immune infiltration and the prognosis of different breast cancer subtypes. Finally, we inferred the prognosis of breast cancer patients after SARS-CoV-2 infection. We found that ACE2 expression decreased significantly in breast cancer, except for basal-like subtype. Decreased ACE2 expression level was correlated with abnormal immune infiltration and poorer prognosis of luminal B breast cancer (RFS: HR 0.76, 95%CI=0.63-0.92, p=0.005; DMFS: HR 0.70, 95%CI=0.49-1.00, p=0.046). The expression of ACE2 was strongly positively correlated with the immune infiltration level of CD8^+^ T cell (r=0.184, p<0.001), CD4^+^ T cell (r=0.104, p=0.02) and neutrophils (r=0.101, p=0.02). ACE2 expression level in the luminal subtype was positively correlated with CD8A and CD8B markers in CD8+ T cells, and CEACAM3, S100A12 in neutrophils. In conclusion, breast tumor tissues might undergo a further decrease in the expression level of ACE2 after SARS-CoV-2 infection, which could contribute to further deterioration of immune infiltration and worsen the prognosis of luminal B breast cancer after SARS-CoV-2 infection.

## INTRODUCTION

Breast cancer occupies the highest incidence of malignant tumors in women and one of the three most common cancers in the world [1–4]. Up to one in nine women may develop breast cancer during lifetime [5]. Breast cancer is now classified into four subtypes according to inherent biological subtypes: HER2 enriched (any HER2^+^), luminal A (HER2^-^/ ER^+^/grade 1,2), luminal B (HER2^-^/ER^+^/ grade 3) and basal like (HER2^-^/ER^-^/PR^-^) [6]. Previously, breast cancer was considered as a tumor type with poor immunogenicity, so the role of immunity in its prognosis was not widely studied. However, in the past few years, some cases of breast cancer have presented to be strongly infiltrated by immune cells [7–9]. The presence of these immune cells has an important predictive value in the prognosis of breast cancer patients.

Since December 2019, more and more pneumonia patients have appeared in Wuhan, Hubei, China, attracting wide concern not only in China, but also in other countries [10]. This previously undefined pneumonia has been named as Coronavirus Disease 19 (COVID-19) by the World Health Organization [11]. Subsequently, scientists isolated novel coronavirus from human airway epithelial cells and named SARS-CoV-2 [12]. The genome analysis of this novel coronavirus showed that the sequence homology was approximately 80-90% similar to original SARS-CoV. ACE2 has been proved to be the receptor of SARS-CoV and NL63 [13]. Recent studies have found that ACE2 is also a receptor binding site for SARS-CoV-2 [14]. Interestingly, ACE2 has been found to be correlated with tumor cell growth and metastasis of pancreatic cancer, breast cancer and colon cancer by inhibiting angiogenesis [15]. However, the potential role of abnormal ACE2 expression correlated with immune infiltration after SARS-CoV-2 infection in the prognosis of breast cancer is still ambiguous.

In the present study, we first analyzed the ACE2 expression level in breast cancer, and its relationship with prognosis in different subtypes of breast cancer. After that, the association between ACE2 and the intensity of immune infiltration in breast cancer was explored. Then we further verified the relationship from the level of immune markers and found that the intensity of immune infiltration was related to the prognosis of breast cancer. Finally, we found the change of ACE2 expression in cells and animal tissues after SARS-CoV infection by analyzing GEO database. Our findings indicated the potential mechanism of immune infiltration mediated by ACE2 in the prognosis of breast cancer after SARS-CoV-2 infection.

## RESULTS

### Differential analysis of ACE2 expression in breast cancer

**Figure 1:**
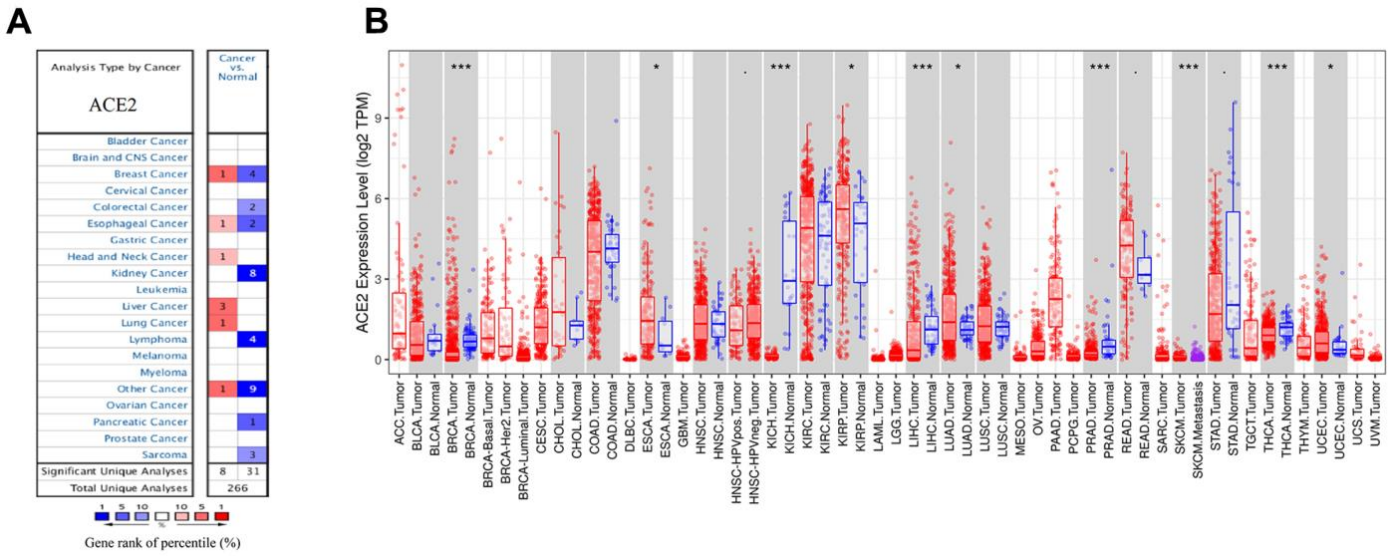
**The expression levels of ACE2 in different cancers.** (**A**) ACE2 in breast cancer compared to normal tissues in the Oncomine database. (**B**) ACE2 expression level of breast cancer and its different subtype in the TCGA database were detected by TIMER (*p<0.05, **p<0.01, ***p<0.001).

Oncomine and TCGA databases were used to analyze the differential mRNA expression of ACE2 between breast cancer and normal tissues. 2,320 samples were finally enrolled in bioinformatic analysis. In Oncomine database, the results indicated that five datasets met our screening criteria (Figure 1A). The study from Finak et al. reported that the ACE2 expression level in invasive breast carcinoma was higher than normal tissue. The other four datasets were analyzed for different subtypes of breast cancer. The analysis from datasets by Ma et al. showed significant lower expression of ACE2 in invasive ductal breast carcinoma than normal tissue. Three other datasets from TCGA showed lower expression in invasive ductal breast carcinoma, invasive lobular breast carcinoma and intraductal cribriform breast adenocarcinoma compared to normal tissues respectively. After that, we further studied the differential ACE2 expression data of breast cancer in TCGA database and found the expression of ACE2 was significantly lower in breast cancer (Figure 1B). In summary, the ACE2 expression level in breast cancer was different due to intrinsic biologic subtypes, most of which were lower than that in normal tissues except for basal-like subtype.

### ACE2 and prognosis of breast cancer

**Figure 2:**
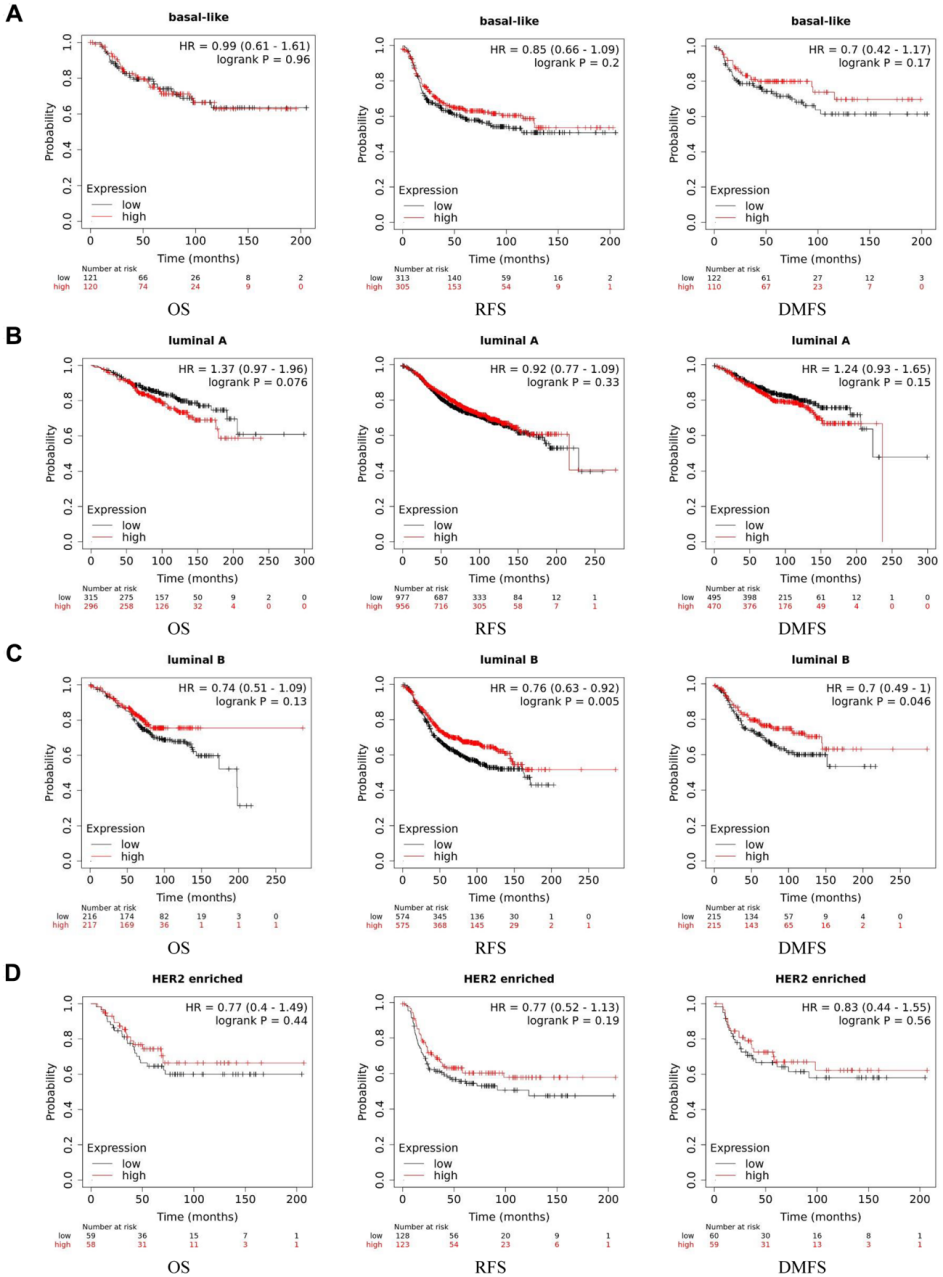
**Kaplan-Meier survival curves of ACE2 expression level in different subtypes of breast cancer.** (**A**) Basal-like, (**B**) luminal A, (**C**) luminal B and (**D**) HER2 enriched. OS, overall survival; RFS, recurrence-free survival; DMFS, distant metastasis-free survival.

We examined the prognostic significance by Kaplan Meier plotter, which covered three major databases: GEO, EGA and TCGA. Considering the different expression level of basal-like subtype, we analyzed the four subtypes separately. We found that in luminal B breast cancer, higher expression level of ACE2 was corelated with better prognosis (RFS: HR 0.76, 95%CI=0.63-0.92, p=0.005; DMFS: HR 0.70, 95%CI=0.49-1.00, p=0.046). While in enriched HER2, luminal A and basal-like subtype of breast cancer, no significant correlation was observed between the expression of ACE2 and the prognosis of breast cancer (Figure 2).

### ACE2 is correlated with immune infiltration in breast cancer

**Figure 3:**
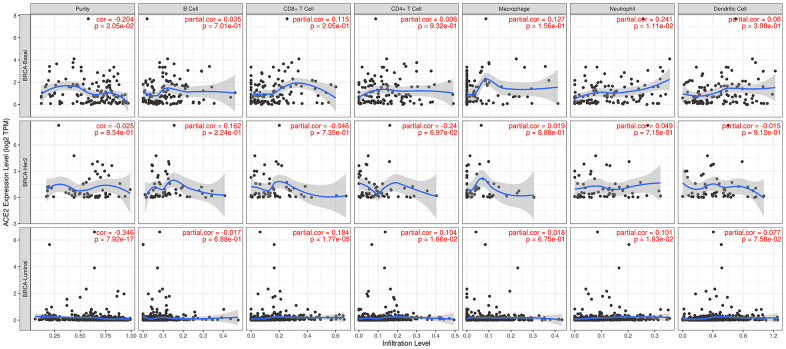
**Correlation between ACE2 expression and immune infiltration in different subtypes of breast cancer.** In basal-like breast cancer, ACE2 expression level was positively correlated with neutrophil. While in luminal subtype of breast cancer, the ACE2 expression was strongly positively correlated with the immune infiltration level of CD8+ T cell, CD4+ T cell and neutrophil.

Recent studies have reported that breast cancer was strongly correlated with immune infiltration, which played a potential role in the prognostic and predictive value. Therefore, we performed analyses in different subtypes of breast cancer on the correlation between transcription level of ACE2 and immune infiltration level by TIMER. Results were shown in Figure 3.

In basal-like breast cancer, ACE2 expression level was positively correlated with neutrophil (r=0.241, p=0.01). While in luminal subtype of breast cancer, the ACE2 expression was strongly positively correlated with the immune infiltration level of CD8^+^ T cell (r=0.184, p<0.001), CD4^+^ T cell (r=0.104, p=0.02) and neutrophil (r=0.101, p=0.02). In HER2 enriched subtype of breast cancer, we found no significant correlation between ACE2 and immune cells.

### ACE2 is related to immune cell type markers

Then we tried to verify the correlation between ACE2 and immune infiltration from level of immune cell type markers. We used TIMER database to analyze the correlation between ACE2 and various immune cell type markers in different subtypes of breast cancer, including B cells, neutrophils, CD8^+^ T cells, dendritic cells, macrophages, NK cells, Th1 cells, Treg and monocytes.

The results showed that ACE2 expression level in the luminal subtype was positively correlated with CD8A and CD8B markers in CD8^+^ T cells, and CEACAM3, S100A12 in neutrophils (Table 1). We also found that ACE2 expression level in the basal subtype was positively correlated with SIGLEC5 and CSF3R markers in neutrophils. These relationships did not change even after adjusting for tumor purity and age. This further proves that the ACE2 expression is related to immune infiltration in breast cancer.

**Table 1:** Correlation analysis between ACE2 expression and different immune cell type markers.

	**Gene markers**	**Basal-like**							**Luminal**							**HER2 enriched**					
		**None**		**Purity**		**Age**			**None**		**Purity**		**Age**			**None**		**Purity**		**Age**	
		**COR**	**P**	**COR**	**P**	**COR**	**P**		**COR**	**P**	**COR**	**P**	**COR**	**P**		**COR**	**P**	**COR**	**P**	**COR**	**P**
B cells	FCRL2	0.044	6.0E-01	-0.091	3.1E-01	0.049	5.7E-01		0.227	1.3E-08	0.111	9.5E-03	0.212	1.3E-07		0.145	2.4E-01	-0.006	9.7E-01	0.142	2.7E-01
	CD19	0.088	3.0E-01	-0.032	7.2E-01	0.093	2.8E-01		0.230	7.4E-09	0.115	7.4E-03	0.218	5.6E-08		0.080	5.2E-01	-0.090	5.0E-01	0.050	7.0E-01
	MS4A1	0.029	7.3E-01	-0.121	1.7E-01	0.041	6.3E-01		0.249	3.5E-10	0.125	3.6E-03	0.241	1.7E-09		0.110	3.8E-01	-0.035	7.9E-01	0.069	5.9E-01
CD8+ T cells	CD8A	0.124	1.5E-01	0.057	5.3E-01	0.120	1.6E-01		0.244	8.0E-10	0.132	2.0E-03	0.234	5.6E-09		-0.087	4.9E-01	-0.173	2.0E-01	-0.079	5.4E-01
	CD8B	0.126	1.4E-01	0.084	3.5E-01	0.133	1.2E-01		0.262	4.1E-11	0.158	2.2E-04	0.253	2.4E-10		-0.067	5.9E-01	-0.177	1.8E-01	-0.067	6.0E-01
Neutrophils	FCGR3B	0.159	6.0E-02	0.145	1.0E-01	0.155	6.8E-02		0.085	3.6E-02	0.070	1.0E-01	0.091	2.6E-02		0.264	3.1E-02	0.240	7.0E-02	0.266	3.5E-02
	CEACAM3	0.073	3.9E-01	-0.058	5.2E-01	0.076	3.8E-01		0.195	1.1E-06	0.131	2.3E-03	0.194	1.4E-06		0.223	7.0E-02	0.252	5.7E-02	0.226	7.6E-02
	SIGLEC5	0.232	5.8E-03	0.186	3.6E-02	0.225	7.9E-03		0.065	1.1E-01	-0.021	6.2E-01	0.060	1.4E-01		-0.088	4.8E-01	-0.138	3.0E-01	-0.118	3.6E-01
	FPR1	0.171	4.4E-02	0.117	1.9E-01	0.165	5.3E-02		0.148	2.2E-04	0.042	3.3E-01	0.143	4.0E-04		-0.112	3.7E-01	-0.180	1.8E-01	-0.122	3.4E-01
	CSF3R	0.260	1.9E-03	0.242	5.9E-03	0.253	2.6E-03		0.059	1.5E-01	0.028	5.2E-01	0.065	1.1E-01		-0.117	3.5E-01	-0.126	3.5E-01	-0.155	2.3E-01
	S100A12	0.129	1.3E-01	0.107	2.3E-01	0.126	1.4E-01		0.206	2.6E-07	0.164	1.3E-04	0.211	1.6E-07		0.203	9.9E-02	0.149	2.6E-01	0.158	2.2E-01
Macrophages	CD68	0.168	4.8E-02	0.095	2.9E-01	0.158	6.4E-02		0.063	1.2E-01	-0.028	5.2E-01	0.060	1.4E-01		0.063	6.1E-01	-0.024	8.6E-01	0.045	7.3E-01
	CD84	0.135	1.1E-01	0.079	3.8E-01	0.128	1.3E-01		0.135	8.1E-04	0.036	4.0E-01	0.129	1.4E-03		0.028	8.2E-01	0.025	8.5E-01	-0.026	8.4E-01
	CD163	0.185	2.8E-02	0.148	9.6E-02	0.176	3.9E-02		0.110	6.2E-03	0.032	4.5E-01	0.110	6.5E-03		0.008	9.5E-01	-0.078	5.6E-01	-0.021	8.7E-01
	MS4A4A	0.228	6.9E-03	0.189	3.3E-02	0.219	9.5E-03		0.161	6.2E-05	0.062	1.5E-01	0.158	9.2E-05		-0.017	8.9E-01	-0.116	3.9E-01	-0.016	9.0E-01
Dendritic cells	CD209	0.103	2.3E-01	0.023	8.0E-01	0.099	2.5E-01		0.195	1.0E-06	0.089	3.8E-02	0.188	3.2E-06		0.037	7.6E-01	-0.045	7.4E-01	0.041	7.5E-01
NK cells	KIR3DL3	0.012	8.9E-01	-0.032	7.2E-01	0.020	8.1E-01		0.101	1.2E-02	0.052	2.2E-01	0.075	6.5E-02		-0.045	7.2E-01	-0.118	3.8E-01	-0.053	6.8E-01
	NCR1	0.143	9.1E-02	0.085	3.4E-01	0.139	1.0E-01		0.178	9.2E-06	0.097	2.4E-02	0.173	1.8E-05		-0.073	5.6E-01	-0.111	4.1E-01	-0.073	5.7E-01
Th1 cells	TBX21	0.160	5.9E-02	0.087	3.3E-01	0.156	6.7E-02		0.244	8.0E-10	0.132	2.1E-03	0.237	3.7E-09		-0.041	7.4E-01	-0.122	3.6E-01	-0.050	7.0E-01
Treg	FOXP3	0.119	1.6E-01	0.033	7.1E-01	0.120	1.6E-01		0.191	1.9E-06	0.086	4.4E-02	0.185	4.6E-06		-0.073	5.6E-01	-0.158	2.4E-01	-0.048	7.1E-01
	CCR8	0.144	9.0E-02	0.120	1.8E-01	0.142	9.5E-02		0.121	2.6E-03	0.062	1.5E-01	0.119	3.4E-03		-0.226	6.6E-02	-0.312	1.7E-02	-0.258	4.1E-02
Monocyte	C3AR1	0.224	7.8E-03	0.174	5.0E-02	0.214	1.1E-02		0.075	6.4E-02	-0.026	5.5E-01	0.070	8.3E-02		-0.050	6.9E-01	-0.092	4.9E-01	-0.061	6.4E-01
	CD86	0.212	1.2E-02	0.160	7.2E-02	0.204	1.6E-02		0.102	1.1E-02	-0.010	8.2E-01	0.099	1.4E-02		-0.066	6.0E-01	-0.143	2.9E-01	-0.069	5.9E-01
	CSF1R	0.206	1.5E-02	0.127	1.5E-01	0.200	1.8E-02		0.105	8.8E-03	-0.051	2.4E-01	0.096	1.8E-02		-0.038	7.6E-01	-0.101	4.5E-01	-0.063	6.3E-01

NK cells, Natural killer cells; Th 1 cells, type I helper T cells; Treg, regulatory T cells; COR, r value of Spearman’s correlation; Purity, correlation adjusted by purity; Age correlation adjusted by age.

### Prognostic analysis of ACE2 expression in breast cancer based on immune cells

We have previously shown that ACE2 expression level was positively correlated with immune filtration in basal-like and luminal subtype of breast cancer, and ACE2 was corelated with prognosis in luminal B breast cancer. Based on these, we may conjecture that immune infiltration plays a potential role in the prognosis of luminal B breast cancer.

**Figure 4:**
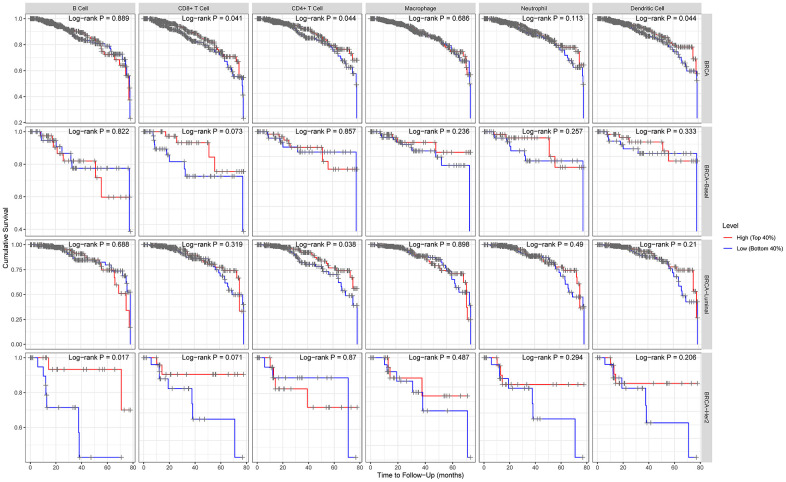
**Comparison of survival curves of the high and low expression of ACE2 in different subtypes of breast cancer based on immune cells subgroups.** For luminal subtype of breast cancer, lower expression level of ACE2 was related to poorer prognosis in enriched CD8+ T cells. The results for HER2 enriched subtype of breast cancer indicated that lower expression of ACE2 was related to poorer prognosis in enriched B cells.

The Kaplan Meier plotter was further used for survival analysis based on related immune cells subgroup (Figure 4). Overall, we found that lower expression level of ACE2 was related to poorer prognosis of breast cancer in enriched CD8^+^ T cells (p=0.04), enriched CD4^+^ T cells (p=0.04) and enriched dendritic cells (p=0.04). In addition, for luminal subtype of breast cancer, lower expression level of ACE2 was related to poorer prognosis in enriched CD8^+^ T cells (p=0.038), which was consistent with the above analysis. The results for HER2 enriched subtype of breast cancer indicated that lower expression of ACE2 was related to poorer prognosis in enriched B cells (p=0.017). However, we found no significant correlation between ACE2 expression level and the prognosis of basal-like breast cancer. These results confirmed that lower ACE2 expression in luminal B breast cancer may worsen prognosis partially through immune infiltration.

### SARS-CoV-2 infection may decrease the expression of ACE2

**Figure 5:**
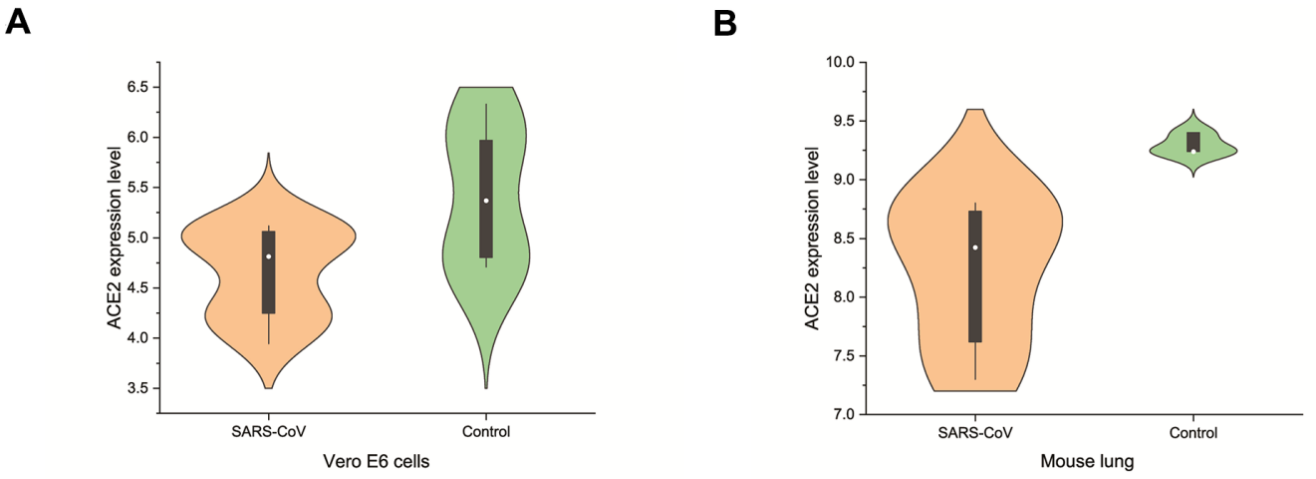
**Changes of ACE2 after SARS-CoV infection.** SARS-CoV reduced the expression levels of ACE2 in (**A**) Vero E6 cells and (**B**) mouse lungs.

In the pathogenesis of SARS-CoV as well as SARS-CoV-2, ACE2 is not only a receptor for viral entry into the host, but also protects against lung injury. It is meaningful to investigate the expression of ACE2 in breast cancer after SARS-CoV-2 infection. SARS-CoV-2 and SARS-CoV spinous proteins share 76.5% homology in amino acid sequence. The expression level change of ACE2 in cells or animals infected with SARS-CoV can be used as a reference for SARS-CoV-2. We analyzed GSE30589 and GSE52920 datasets from GEO database, which investigated the change of ACE2 expression in Vero E6 cells and mice lung after SARS-CoV infection. The results showed that the expression of ACE2 in animal cells and mouse lung decreased significantly compared with the control group (Figure 5). These data suggest that SARS-CoV-2 infection may decrease the expression level of ACE2 in breast cancer.

## DISCUSSION

In present study, we used bioinformatics methods to first analyze changes in the expression level of ACE2 mRNA in breast cancer. Then we studied the potential role of ACE2 in the prognosis of different subtypes of breast cancer, and then found that the ACE2 expression was related to immune infiltration, and further analyzed the relationship between immune infiltration and the prognosis of different subtypes. Finally, we studied the prognosis of breast cancer patients after SARS-CoV-2 infection.

We found that the ACE2 expression level was lower than normal tissues except for basal like subtypes in breast cancer. Then we analyzed the relationship between ACE2 in four subtypes of breast cancer and prognosis by Kaplan Meier plotter. The higher expression of ACE2 was correlated with a better prognosis in luminal B-type breast cancer (RFS: HR 0.76, 95% CI = 0.63-0.92, p = 0.005; DMFS: HR 0.70, 95% CI = 0.49-1.00, p = 0.046). After that, we analyzed the relationship between ACE2 and immune infiltration. In the luminal breast cancer, ACE2 is related to the immune infiltration of CD8^+^ T cells (r = 0.184, p < 0.001), CD4^+^ T cells (r = 0.104, p = 0.02) and neutrophils (r = 0.101, p = 0.02). Then, we further verified the correlation between ACE2 and immune infiltration from the level of immune cell type markers. The results showed that ACE2 was positively related with CD8A and CD8B in CD8 + T cells and CEACAM3 and S100A12 in neutrophils (Table 1). Even after adjusting for purity and age, these relationships still exist. In addition, the lower expression level of ACE2 was related to the poor prognosis in enriched CD8^+^ T cells (p = 0.038). These results indicate that the low expression of ACE2 in luminal B breast cancer is related to the worse prognosis partially because of immune infiltration. Finally, we analyzed the expression of ACE2 in Vero E6 cells and lung of mice infected with SARS-CoV from GEO database. The results showed that compared with control group, ACE2 in Vero E6 cells and mice lungs was significantly reduced (Figure 5). These data suggest that SARS-CoV-2 infection may reduce ACE2 expression in breast cancer. The prognosis of luminal B breast cancer would be further deteriorated, and immune infiltration might be one of the mechanisms for prognosis deterioration.

The incidence of breast cancer increases with age [16]. The intrinsic subtype and immune infiltration intensity are considered to be the important biomarkers for risk stratification and prognosis prediction of breast cancer patients [17, 18]. ACE2 belongs to renin-angiotensin system, which plays a critical role in heart and vessels [19]. The metabolic product of this enzyme has the function of vasodilation, antiproliferation and anti-fibrosis [20]. Recently, it has been found that RAS members are involved in various biological processes in different tumors [21]. It is reported that AngII accelerates tumor migration, proliferation, angiogenesis by activating AT1R, while in lung cancer, the activation of AT2R accelerates tumor proliferation and angiogenesis. ACE2 and Ang (1-7) have been proved to inhibit the metastasis progression of prostate cancer and lung cancer. ACE2 is also reported to have potential antitumor effects in a variety of malignant diseases, including liver, lung and prostate cancer [22]. Recently, a study revealed lower ACE2 expression in breast cancer cells compared to normal tissues, and the low ACE2 level contributed to the deterioration of prognosis, indicating that ACE2 may act as a beneficial effect on breast cancer [23], which is consistent with the conclusion of our study. In addition, according to previous studies, ACE2 could also inhibit breast cancer cell migration and proliferation. The above results indicate that ACE2 has potential antitumor effect and inhibits the progression of breast cancer.

Tumor angiogenesis is generally mediated by angiogenic factors [24]. VEGFa might play a key role in the process of inhibiting angiogenesis by ACE2 in breast cancer. It was reported that ACE2 decreased the VEGFa expression of breast cancer cells, inhibiting phosphorylation of ERK1/2, suggesting that ERK signaling pathway mediated by ACE2 participated in the regulation of VEGFa [25]. Research confirmed VEGFa in tumor cells would be bind to VEGFR2 on the membrane of adjacent endothelial cells. This binding accelerating phosphorylating and activating VEGFR2, which further leads to the phosphorylation and activation of the ERK signaling pathway [26]. After that, phosphorylation and activation of MEK1/2 were started through cascade reaction from ERK pathway, promoting phosphorylation and activation of ERK1/2 [27]. As a result, nuclear translocation could accelerate HUVEC differentiation, migration and proliferation, which ultimately promotes angiogenesis [28]. Therefore, the mechanism of ACE2 to inhibit angiogenesis of breast cancer might be related to VEGFa/VEGFR2/ERK pathway. In this study, we found that ACE2 may affect the prognosis of breast cancer through the new mechanism-immune infiltration, which provides a direction for further research in the future. However, some limitations do exist, such as the urgent need for experimental verification of the results of bioinformatics analysis in the study.

## CONCLUSIONS

ACE2 expression decreased significantly in breast cancer, except for basal-like subtype. Decreased ACE2 expression level was correlated with abnormal immune infiltration and worse prognosis in luminal B breast cancer. Tumor tissues might undergo a further decrease in the expression level of ACE2 after SARS-CoV-2 infection, which could produce further deterioration of immune infiltration and worsen the prognosis of luminal B breast cancer after SARS-CoV-2 infection.

## MATERIALS AND METHODS

### Differential analysis of ACE2 expression

Oncomine (https://www.oncomine.org/) [29] and TIMER (http://timer.cistrome.org/) [30] database were used to explore the ACE2 expression in different biological intrinsic subtypes in breast cancer and the threshold was set at p-value =0.01, 1.5-fold change, top 10% of gene rank, data type of mRNA.

### Survival analysis

Kaplan-Meier plotter (http://kmplot.com/) [31] was used to explore the ACE2 expression level and prognosis in different biological intrinsic subtypes in breast cancer. The OS, RFS and DMFS were calculated in each subtype respectively. Redundant samples and biased arrays were removed before analysis. The log-rank p <0.05 in Kaplan-Meier plotter was considered statistically significant.

After that, we used TIMER (http://timer.cistrome.org/) database to explore the immune infiltration level (including CD8^+^ T cells, B cells, CD4^+^ T cells, Dendritic cells Neutrophils and Macrophages) and the prognosis of breast cancer. Then we further analyzed the immune cell markers (FCRL2, CD19, MS4A1 from B cells, CD8A, CD8B from CD8+ cells, FCGR3B, CEACAM3, SIGLEC5, FPR1, CSF3R, S100A12 from neotrophils, CD68, CD84, CD163, MS4A4A from macrophages, CD209 from dendritic cells, KIR3DL3, NCR1 from NK cells, TBX21 from Th1 cells, FOXP3, CCR8 from Treg cells and C3AR1, CD86, CSF1R from monocytes) and ACE2 expression level and the correlation between immune infiltration and prognosis of different subtypes of breast cancer.

### ACE2 expression change after infection using microarray data analysis

We used two datasets (GSE30589 and GSE52920) from Gene Expression Omnibus to explore the ACE2 expression level change after SARS-CoV infection. The GSE30589 database contains genome-wide expression data between SARS-CoV-infected, and SARS-CoV-ΔE-infected and mock-infected cells based on Affymetrix microarrays. For each type of sample, three biological replicates were independently hybridized. While the GSE52920 database used Agilent-028005 SurePrint G3 Mouse GE 8x60K Microarray contains genome-wide expression data between SARS-CoV-wt, SARS-CoV-mutPBM and Mock infected mouse lungs. Three biological replicates were independently hybridized.

### Statistical analysis

We used the log rank test for survival analysis. The correlation of gene expression was calculated by Spearman correlation test. Student t test was used for testing statistical difference between two independent variables. All p values less than 0.05 were considered statistically significant. The violin plot was drawn by Origin 2020 software.
